# SUSTAINING STAR-VA: EVALUATING SYSTEMIC OUTCOMES AND TEAM STRATEGIES FOR MANAGING BEHAVIOR SYMPTOMS OF DEMENTIA

**DOI:** 10.1093/geroni/igz038.2350

**Published:** 2019-11-08

**Authors:** Kim Curyto, Kimberly Van Haitsma

**Affiliations:** 1 Center for Integrated Healthcare, VA Western New York Healthcare System, Batavia, New York, United States; 2 Penn State, University Park, Pennsylvania, United States

## Abstract

The Veterans Health Administration (VHA) has invested in the implementation and evaluation of STAR-VA, a Veteran-centered, interprofessional intervention for managing behavioral symptoms of dementia (BSD), with 86 Community Living Center (CLC nursing home) teams between 2013 and 2018. Results of a VHA Quality Enhancement Research Initiative (QUERI) partnered evaluation project are presented, including a multi-site interprofessional network created to collaborate on evaluation of the longitudinal impact of STAR-VA on CLC Veteran- and site-level outcomes, and to determine factors associated with sustained implementation and positive outcomes. The development and validation of a Minimum Data Set quality indicator of behavior symptoms of dementia (BSD) is presented. Characteristics of STAR-VA trained and untrained CLCs are presented, along with facilitators and barriers to sustained program implementation. Findings support the effectiveness of implementation of STAR-VA on decreased use of psychotropic medication. Qualitative outcomes demonstrate importance of having the appropriate staff, positive team relationships, supportive usual routines, and culture as critical for STAR-VA sustainability efforts. Results emphasize the importance of using a valid, routine measure of BSD to provide feedback to CLC teams, and to develop a sustainability intervention focused on addressing reported barriers to program sustainability through interprofessional networks. Both qualitative and quantitative outcomes will inform development and recommendations for an implementation and evaluation strategy for an outcome-driven, tailored intervention to support CLC teams in sustaining STAR-VA and to improve poor-performer and maintain high-performer outcomes. The potential benefit of our findings for other interprofessional behavioral nursing home interventions will be discussed.

